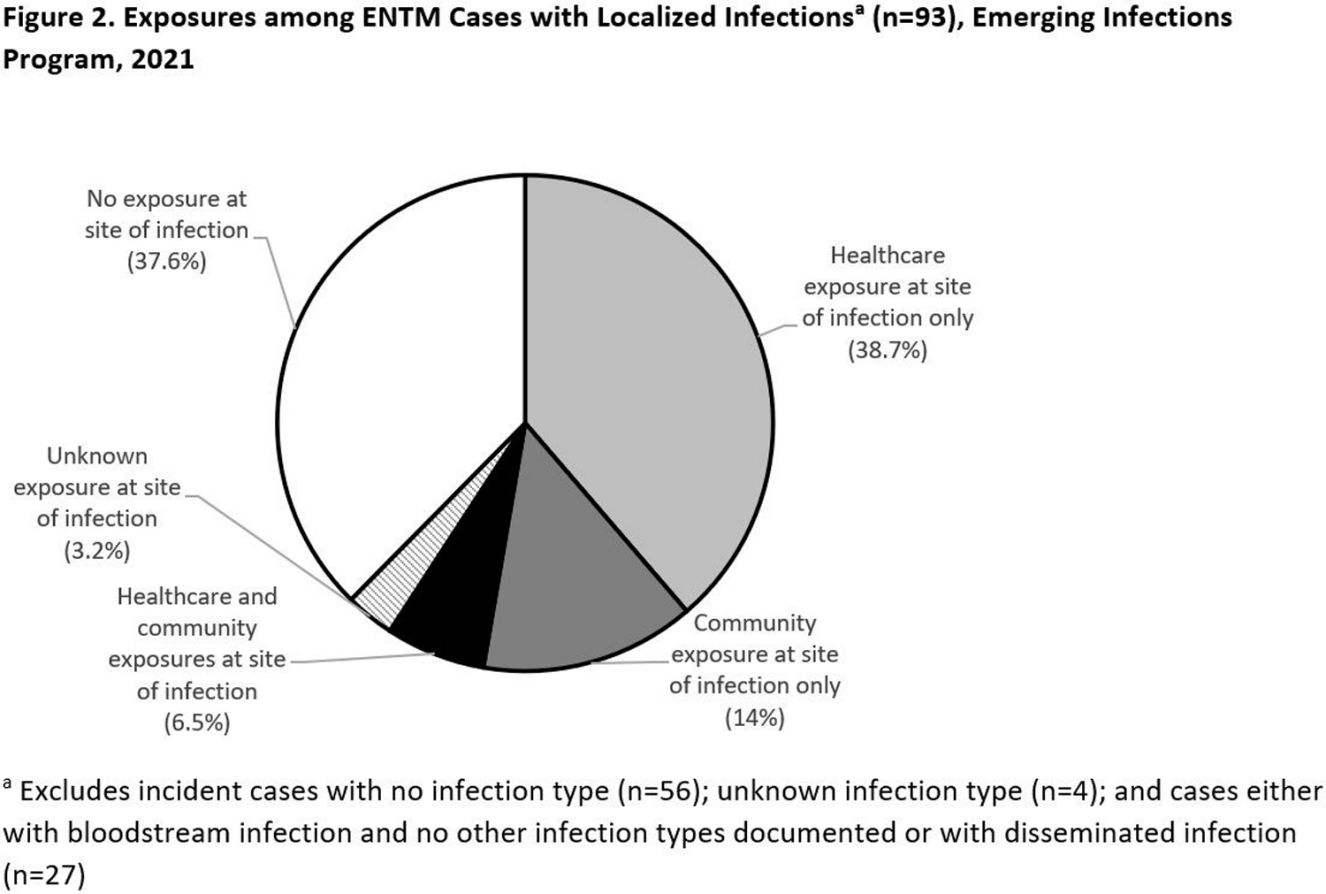# Epidemiology of Extrapulmonary Nontuberculous Mycobacterial Disease – 4 Emerging Infection Program Sites, 2021

**DOI:** 10.1017/ash.2024.231

**Published:** 2024-09-16

**Authors:** Rebecca Byram, Kelly Jackson, Christopher Czaja, Helen Johnston, Devra Barter, Ruth Lynfield, Nathan Centurion, Laura Tourdot, Ghinwa Dumyati, Christopher Myers, Rebecca Pierce, Nadege Charles Toney, Adel Mansour, Shelley Magill, Isaac See

**Affiliations:** Centers for Disease Control and Prevention; Chenega Enterprise Systems and Solutions; Colorado Department of Public Health and Environment; Minnesota Department of Health; University of Rochester Medical Center; NY Emerging Infections Program; Oregon Emerging Infections Program

## Abstract

**Background:** Extrapulmonary nontuberculous mycobacteria (ENTM) infections are difficult to treat and often require prolonged therapy or surgery. Few population-based studies describe ENTM epidemiology, though well-known healthcare-associated outbreaks have occurred. Using the first year of multi-site ENTM surveillance, we characterized rates and how frequently ENTM infections may be related to healthcare. **Methods:** CDC’s Emerging Infections Program conducted active, laboratory- and population-based surveillance for ENTM cases in 4 sites (Colorado [5 counties], Minnesota [statewide], New York [1 county], and Oregon [statewide]) in 2021. An incident ENTM case was NTM isolation from a non-pulmonary specimen, excluding stool or rectal swabs, in a resident of the surveillance area without either medical record documentation of prior ENTM infection or isolation of ENTM in the prior 12 months. Demographic, clinical, information on selected healthcare and community exposures, and laboratory data were collected via medical record review. We calculated incidence per 100,000 population using U.S. Census population estimates and performed descriptive analyses. **Results:** A total of 180 incident ENTM cases were reported in 2021. The crude annual incidence rate was 1.3 per 100,000 persons. Incidence increased with age (from 0.95 per 100,000 among 0–17 year-olds to 2.65 per 100,000 among persons ≥65), ranged from 0.8 among non-Hispanic Asian persons to 1.6 per 100,000 in non-Hispanic Black persons, and was similar among males (1.3 per 100,000) and females (1.4 per 100,000; Figure 1). Mycobacterium avium complex (64 [35.6%]) was the most frequently isolated species group, followed by Mycobacterium chelonae complex (31 [17.2%]). Skin and soft tissue infections were the most frequent infection type (37 [20.6%]); 27 cases (15.0%) were associated with disseminated and/or only bloodstream infection, and 56 cases (31.1%) had no infection type documented. Among 93 cases with localized ENTM infections (i.e., infections that were not disseminated and/or only bloodstream infections), 38.7% had only healthcare-related exposures, 14% had only community-related exposures and 6.5% had both exposure types at the site of infection (Figure 2). Healthcare-related exposures at the infection site included surgery (23.7%), injection/infusion (21.5%), and medical devices (18.3%). The most frequent community-related exposure at the infection site was trauma (17.2%). Only one case was part of a known outbreak, which was healthcare-associated. **Conclusions:** ENTM infections are relatively rare, but nearly half of patients with localized ENTM infections had prior healthcare-related exposures. This indicates that the burden of ENTM infections related to healthcare may be much larger than what has been suggested from reported outbreaks.

**Figure:**
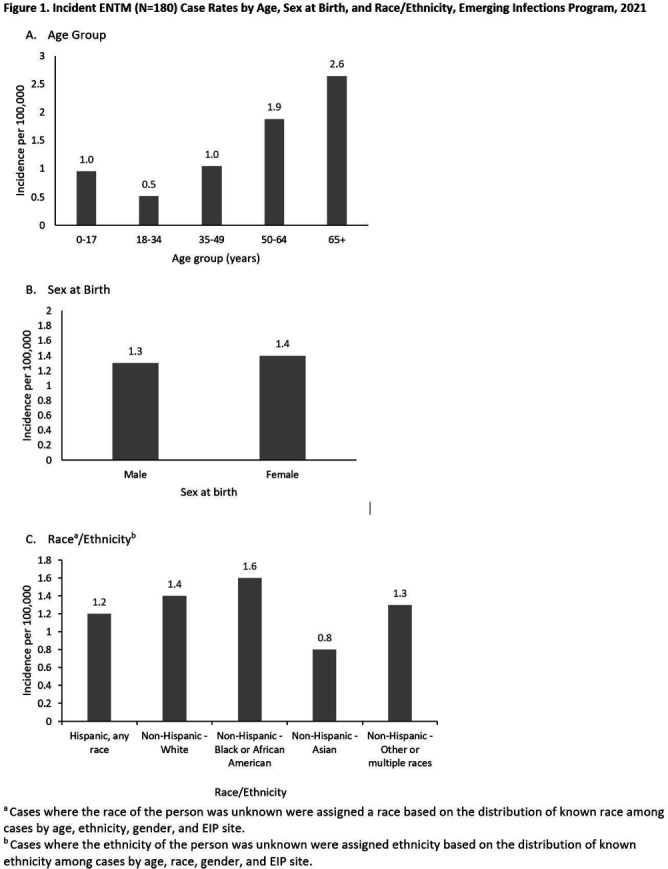

**Figure:**